# Unripe Black Raspberry (*Rubus coreanus* Miquel) Extract and Its Constitute, Ellagic Acid Induces T Cell Activation and Antitumor Immunity by Blocking PD-1/PD-L1 Interaction

**DOI:** 10.3390/foods9111590

**Published:** 2020-11-02

**Authors:** Ji Hye Kim, Young Soo Kim, Tae In Kim, Wei Li, Jeong-Geon Mun, Hee Dong Jeon, Ji-Ye Kee, Jang-Gi Choi, Hwan-Suck Chung

**Affiliations:** 1 Korean Medicine (KM)-Application Center, Korea Institute of Oriental Medicine (KIOM), Dong-gu, Daegu 41062, Korea; jkim2903@kiom.re.kr (J.H.K.); yskim527@kiom.re.kr (Y.S.K.); tikim@kiom.re.kr (T.I.K.); liwei1986@kiom.re.kr (W.L.); 2 Department of Oriental Pharmacy, College of Pharmacy, Wonkwang-Oriental Medicines Research Institute, Wonkwang University, 460 Iksandae-ro, Iksan, Jeonbuk 54538, Korea; wjdrjs92@daum.net (J.-G.M.); alen0707@naver.com (H.D.J.); keejy@wku.ac.kr (J.-Y.K.)

**Keywords:** black raspberry, ellagic acid, antitumor immunity, T cell function, programmed cell death protein 1, programmed death-ligand 1

## Abstract

*Rubus coreanus* Miquel (*R. coreanus*) is a unripen fruit of black raspberry native to eastern Asia. It is used as traditional oriental medicine and supplementary foods for centuries. Previous studies have shown that the *R. coreanus* extract (RCE) and its main constitute ellagic acid possess diverse biological activities. However, the effects of RCE on antitumor immunity and T cell function were not fully understood. The present study describes the anti-tumor effect of RCE in humanized PD-1 mice by blocking PD-1/PD-L1 interaction. Competitive enzyme-linked immunosorbent assay (ELISA) and pull down assay were performed to elucidate the binding properties of RCE in vitro. Cellular PD-1/PD-L1 blockade activities were measured by T cell receptor (TCR)-induced nuclear factor of activated T cells-luciferase activity in co-cultured cell models with PD-1/NFAT Jurkat and PD-L1/aAPC CHO-K1 cells. The in vivo efficacy of RCE was confirmed in humanized PD-1 mice bearing MC38 colorectal tumor. RCE and ellagic acid dose-dependently block the binding of PD-1 to PD-L1. Moreover, oral administration of RCE showed the potent anti-tumor activity similar to anti-PD-1 antibody. The present study suggests that RCE possesses potent anti-tumor effect via PD-1/PD-L1 blockade, and ellagic acid is the main compound in RCE. Thus, we provide new aspects of RCE as an immunotherapeutic agent.

## 1. Introduction

Recent advances in immuno-oncology (I-O) therapy demonstrated the promising clinical evidences of immune checkpoint inhibitors for multiple types of cancers [1]. The programmed cell death protein 1 receptor (PD-1), and its ligand programmed death ligand 1 (PD-L1), are one of most extensively studied immune checkpoints [2]. The PD-1 and PD-L1 are major co-inhibitory receptors that involved in T cell–mediated immunity [3]. The recognition between PD-L1 and PD-1 decreases T cell functions including the proliferation and Interleukin (IL)-2 cytokine release in the tumor microenvironment [4]. Therefore, one of promising strategy for the anti-tumor immunity has been focused on the upregulation of T cell function by blocking PD-1/PD-L1 axis.

Therapeutic antibodies to target PD-1 and/or PD-L1 are considered as promising immunotherapeutic agents against cancers [5]. Additionally, many studies on small molecules have been performed for due to their modulating activities in the PD-1/PD-L1 pathway. These individual inhibitors have their strengths and shortcomings in accordance with their different physiochemical characteristics [6]. Particularly, studies about non-antibody drugs for PD-1/PD-L1 inhibitors have been ongoing in order to surpass disadvantages of antibody drugs such as high costs and immune-related adverse events (irAEs) [7].

*Rubus coreanus* Miquel (*R. coreanus*) is a specie of *Rubus* genus native to eastern Asia. Its common name is black raspberry referred to as “bokbunja” in Korean. The immature fruits of *R. coreanus* have been utilized in traditional medicine for centuries [8]. Previous studies have demonstrated that *R. coreanus* extract (RCE) also exerts diverse biological effects that may be beneficial to human health [9,10]. Many phytochemical constituents of RCE were previously reported, including various phenolic compounds [11]. Especially, ellagic acid is a major phenolic compound of black raspberry fruit known to have a powerful antioxidant and anti-tumor capacities [12]. However, the detailed immunomodulatory mechanism of their action targeting the PD-1/PD-L1 immune checkpoint is not fully understood. Therefore, the present study elucidated whether RCE and its major component, ellagic acid, inhibit the binding of PD-1 to PD-L1 using competitive enzyme-linked immunosorbent assay (ELISA) and cell-based bioassay. Additionally, we also investigated whether RCE can influence the growth of MC38 tumors expressing human PD-L1 in humanized PD-1 mice.

## 2. Materials and Methods

### 2.1. Materials

The human PD-1/PD-L1 competitive enzyme-linked immunosorbent assay (ELISA) kit and antagonist antibody to human PD-L1 (αPD-L1) were purchased from BPS Bioscience Inc. (San Diego, CA, USA). The PD-1/PD-L1 Blockade Bioassay Kit was purchased from Promega Co. (Madison, WI, USA). Antagonist antibody to human PD-1 (αPD-1, pembrolizumab) for animal experiments was purchased from Selleck Chemicals (Houston, TX, USA). Primary antibody for PD-1 (#367402) was from obtained from Biolegend Inc. (San Diego, CA, USA). Primary antibody for PD-L1 (#13684) was from obtained from Cell signaling Technology Inc. (Danvers, MA, USA). All solutions for cell culture including Dulbecco’s Modified Eagle’s Medium (DMEM), RPMI 1640 medium, F-12 Kaighn’s Modification medium, fetal bovine serum (FBS), 0.25% trypsin-ethylenediaminetetraacetic acid (EDTA), and penicillin–streptomycin were purchased from Hyclone Laboratories Inc. (Chicago, IL, USA).

### 2.2. Preparation of RCE

Unripe black raspberries (*R. coreanus*) were provided from the National Development Institute of Korean Medicine (NIKOM, Gyeongsan, Korea). RCE (1.0 kg) were extracted with distilled water under reflux for 2 h (5 L × 3 times). The RCE solution were filtered using filter paper, and freeze-dried to powder (122.0 g) for 5 days. All samples were stored in a desiccator at 4 °C until the experiments.

### 2.3. PD-1/PD-L1 Competitive ELISA

To examine the effect of test samples on PD-1/PD-L1 interaction, PD-1/PD-L1 Competitive ELISA kits were performed by the distributor’s manuals [13]. In a brief, recombinant hPD-L1 protein was diluted at 1 μg/mL in phosphate-buffered saline (PBS, pH 7.4) and then was coated in 96-well white plates at 4 °C overnight. Plates were washed with PBS containing 0.1% Tween (PBS-T) for three times and incubated with blocking solution for 1 h at room temperature (RT). After pretreatment with test inhibitors for 1 h at RT, fifty microliters of 0.5 μg/mL biotinylated hPD-1 was added to the wells, and the plates were further incubated for 2 h at RT. After three washes in PBS-T, 50 μL of 0.2 μg/mL horseradish peroxidase (HRP)-conjugated streptavidin was added to each well, and the plates were incubated for 1 h. After incubation and washing, and relative chemiluminescence was measured on a SpectraMax L Luminometer (Molecular Devices, San Jose, CA, USA).

### 2.4. Cell Culture

Jurkat cells expressing human PD-1 and NFAT reporter gene (PD-1/NFAT Jurkat cells) and CHO-K1 cells expressing human PD-L1 and a T cell receptor activator (PD-L1/aAPC CHO-K1 cells) were purchased from BPS Bioscience (San Diego, CA, USA). PD-1/NFAT Jurkat cells were incubated in RPMI 1640 medium containing 10% heat-inactivated FBS, 1% penicillin, and streptomycin at 37 °C and 5% CO_2_ incubator. PD-L1/aAPC CHO-K1 cells were cultured with DMEM/F-12 medium containing 10% heat-inactivated FBS and 1% penicillin/streptomycin at 37 °C and 5% CO_2_. To maintain the stable cells containing the genetic constructs, cells were cultured in complete medium with Hygromycin B (200 μg/mL) and G418 (1 mg/mL). These antibiotics were removed from the medium during the experiments. MC38 cells expressing human PD-L1 (hPD-L1 MC38 cells) were purchased from Shanghai Model Organisms Center, Inc. (Shanghai, China). The hPD-L1 MC38 cells were maintained in DMEM containing 10% heat-inactivated FBS and 1% penicillin and streptomycin at 37 °C and 5% CO_2_ condition.

### 2.5. Cell Viability Assay

Cell viability was analyzed by Cell Counting Kit-8 (CCK) assay as the distributor’s manuals (Dojindo Molecular Technologies, Inc., Rockville, MD, USA). CCK is one of sensitive colorimetric assay for the determination of cell viability. WST-8 is highly water-soluble tetrazolium salt and is changed by dehydrogenases in cells to a yellow formazan. In a brief, cells with a density of 1 × 10^4^ cells/100 μL were seeded into 96-well plates and incubated overnight at 37 °C. Test inhibitors were added to the wells at the indicated concentrations. After incubation for the indicated time, 10 μL of CCK solution was treated for 2 h at 37 °C. The amount of the formazan dye, converted by cellular dehydrogenases, is soluble in the cell culture medium and it means to the number of living cells. To measure the color density at 450 nm, a microplate reader from Molecular Devices i3 (San Jose, CA, USA) was used.

### 2.6. Cellular PD-1/PD-L1 Blockade Assay

The cellular PD-1/PD-L1 blockade assay was used to measure the potency of test inhibitors blocking the NFAT-luciferase reporter activity in co-cultured cell model system as previously described [14]. In a brief, the 100 μL of PD-L1 aAPC/CHO-K1 cells with a density of 5 × 10^5^ cells /mL were seeded in 96-well plates. A few hours later, PD-L1 aAPC/CHO-K1 cells were co-cultured with 100 μL of PD-1/NFAT Jurkat cells (1 × 10^6^ cells/mL). At the same time, test inhibitors were added into each well and cultured for 24 h at 37 °C. After adding the lysis solution and substrates using the Bio-Glo™ Luciferase Assay kit (Promega Co., Madison, WI, USA), the relative luminescence intensity was detected by a SpectraMax L Luminometer (Molecular Devices, San Jose, CA, USA). Data were calculated by fold to vehicle-treated group.

### 2.7. Cytokine Measurement

The cytokine release of human interleukin (IL)-2 was detected by BD OptEIA ELISA kits (BD Biosciences) according to the supplier’s manuals [15]. In brief, 96-well plates were coated with 100 μL/well of capture antibodies to IL-2 in coating buffer overnight at 4 °C. The plates were washed five times with PBS (pH 7.4) containing 0.05% Tween 20 (PBST; Biosesang, Korea), and then each well was incubated with 200 μL of 10% FBS in PBS at 37 °C for 1 h to block nonspecific protein binding. After five washes with PBST, standards of cytokine and supernatant samples of 100 μL were incubated at RT for 2 h. After washing five times with PBST, the plate was reacted with 100 μL solution containing detection antibodies and SAv-HRP at RT for 1 h and washed seven times with PBST. Then, wells were incubated with 100 μL of tetramethylbenzidine in the dark for 15 min and the reaction was finished by adding 50 μL of 1M H_2_SO_4_. The absorbance was read at 450 nm using a microplate reader. The production levels of IL-2 were calculated from the standard curves in rage from 0 to 1000 pg/mL.

### 2.8. Animals with Experiments

C57BL/6J mice were genetically modified for replacement of endogenous PDCD1 in the mouse to human PDCD1 so that expressing full length human PD-1 protein. Humanized PD-1 mice (C57BL/6 mice expressing human PD-1 protein) were purchased from Shanghai Model Organisms Center, Inc. (Shanghai, China). Humanized PD-1 mice (10 week old, male) were adapted for one week before experiments and freely consumed food and water. Animals were maintained in constant conditions (25 °C) with a 12 h light/dark cycle. All animals were housed under specific pathogen-free (SPF) facilities of Korea Institute of Oriental Medicine (KIOM, Daegu, Korea). All experiments with mice were conducted according to the rules and acceptance of the Institutional Animal Care and Use Committee (IACUC) of KIOM (approval number: KIOM-D-19-003).

### 2.9. In Vivo Evaluation Using hPD-L1 MC38 Tumor Model in Humanized PD-1 Mice

All animals with experiments were anaesthetized by 2% isoflurane inhalation with a 1:1 oxygen using V-1 laboratory animal anesthesia tool from VetEquip Inc. (Livermore, CA, USA). Under anaesthetized condition, hair of both flanks were removed by shaving for precise injection and observation of tumor cells. The hPD-L1 MC38 cells with 1.5 × 10^6^ cells/mL were subcutaneously (s.c.) injected into the right flank of humanized PD-1 mice (3 × 10^5^ cells/200 μL/mouse). Tumor volume was measured twice a week using digital caliper from Hi-Tech Diamond (Westmont, IL, USA). The tumor volume was calculated according to the following formula: (S^2^ × L)/2 (S: short diameter; L: long diameter). When mean tumor volumes were calculated at approximately ~100 mm^3^ at 14 days post injection, humanized PD-1 mice were randomized with four groups (*n* = 6 per group): vehicle-treated group (PBS, 10 mL/kg, q.d., i.g.), anti-PD-1-treated group (ketruda, 5 mg/kg, biwx2, i.p.), low RCE groups (50 mg/kg, q.d., i.g.) and high dose of RCE (100 mg/kg, q.d., i.g.). On days 3, 7, 10, 14, 17, and 21 post injection, mice were intraperitoneally (i.p.) treated with PBS or αPD-1 antibody. Intragastric (i.g.) injection of RCE were given daily. All mice were sacrificed for analyses 21 days after the treatment.

### 2.10. High-Performance Liquid Chromatography (HPLC) Analysis

The content of ellagic acid was analyzed by the HPLC profiles of RCE (5 mg/mL) and standard ellagic acid (15.11 μg/mL) using Alliance e2695 (Waters Corp., Milford, MA, USA) by injecting 10 μL of sample into a Geminin C18 column (5 μm, 250 × 4.6 mm; Phenomenex Inc., Torrance, CA, USA) at an oven temperature of 40 °C. The mobile phase was applied at a flow rate of 1.0 mL/min with a gradient of acetonitrile containing 1% acetic acid (A) and distilled water containing 1% acetic acid (B) as follows: 10% A (0–3 min), 10–55% A (3–33 min), 33–100% A (33–38 min), 100% A (38–39 min). The samples were monitored under UV light at 254 nm.

### 2.11. Preparation of Ellagic Acid–Sepharose 4B Beads

Sepharose 4B powder was purchased from GE Lifesciences (Piscataway, NJ, USA). By adding 1 mM HCl, Sepharose 4B powder (0.3 g) was activated by a previous method with a slight modification [16]. The activated Sepharose 4B beads was conjugated with ellagic acid in coupling solution (0.1 M NaHCO_3_ pH 8.3, and 0.5 M NaCl) at 4 °C overnight with gently rotation. The mixture was washed out with the coupling solution, and then transferred to reaction buffer (0.1 M Tris-HCl buffer, pH 8.3). The uncoupled ellagic acid was washed out with washing solution (0.1 M acetate buffer (pH 4.0), and 0.1 M Tris-HCl buffer (pH 8.0) containing 0.5 M NaCl), and the coupled ellagic acid-conjugated Sepharose 4B beads (EA-Sepharose 4B) were resuspend in PBS.

### 2.12. In Vitro Pull-Down Assays

Recombinant PD-1 or PD-L1 (500 ng) was incubated with EA-Sepharose 4B beads or with Sepharose 4B beads alone (as a control) in reaction buffer (150 mM NaCl, 5 mM EDTA, 50 mM Tris pH 7.5, 1 mM 1,4-dithiothreitol (DTT), 1 μg protease inhibitor mixture, 0.02 mM phenylmethylsulfonyl fluoride (PMSF), 2 μg/mL bovine serum albumin and 0.01% Nonidet P-40) by rotation overnight at 4 °C. After washing the beads five times with washing buffer (150 mM NaCl, 5 mM EDTA, 50 mM Tris pH 7.5, 1 mM DTT, 0.02 mM PMSF, and 0.01% NP-40), and proteins bound to the beads were detected by Western blot.

### 2.13. Western Blot

Proteins bound to the beads were boiled with sample buffer (0.1 M Tris-HCl, 0.2 M DTT, 4% SDS, 0.06% Bromophenol blue, 20% Glycerol) at 95 °C. Separated proteins by SDS-PAGE were transferred to an Immobilon*^®^*-P membrane (Merck Millipore Ltd., Carrigtwohill, County Cork, Ireland). After blocking in 5% nonfat milk in TBS, containing 0.1% Tween 20 (TBST) the membrane was incubated with primary antibodies for PD-1 or PD-L1 at 4 °C overnight. After washing with TBST, an HRP-conjugated secondary antibody was incubated with the membranes and target proteins were detected by Clarity Western ECL solution with peroxide and luminol reagent (Bio-Rad, Hercules, CA, USA).

### 2.14. Statistical Analysis

The data were presented as mean ± standard error (S.E.). Differences in the mean value between the treatment and untreated group were compared by one-way ANOVA analysis with Dunnett’s post-hoc test or two-way ANOVA with Bonferroni post-hoc test. *p*-values < 0.05 were considered significant and indicated using asterisks. The statistical analysis was conducted by GraphPad Prism program version 5.02 (La Jolla, CA, USA)

## 3. Results

### 3.1. Effects of RCE on the PD-1/PD-L1 Protein Interaction and T Cell Function In Vitro

**Figure 1:**
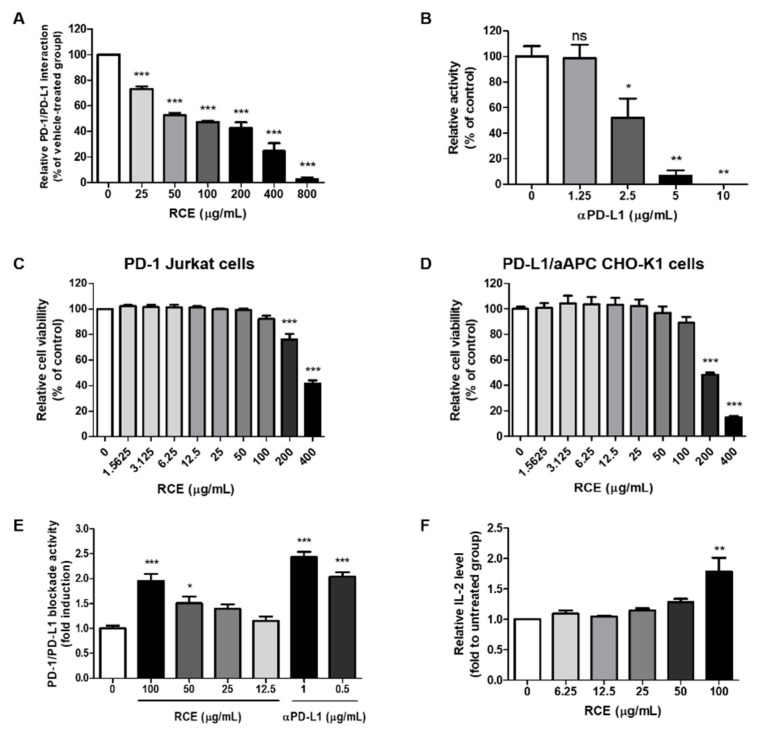
Effect of *Rubus coreanus* extract (RCE) on programmed cell death protein 1 (PD-1)/PD-1 ligand-1 (PD-L1) protein interaction using competitive enzyme-linked immunosorbent assay (ELISA) or cell-based assay. (**A**,**B**) The effect of RCE on the binding of PD-1 and PD-L1 in vitro using a competitive ELISA assay. (**C**,**D**) The effect of RCE on cell viability of PD-1/NFAT Jurkat cells (**C**) and PD-L1/aAPC CHO-K1 cells (**E**) using the Cell Counting Kit-8 (CCK) assay. Each of the cells were treated according to the indicated concentrations of RCE for 24 h. (**E**) The effect of RCE on cellular PD-1/PD-L1 blockade activity. PD-1/NFAT Jurkat effector cells and PD-L1/aAPC CHO-K1 target cells were co-cultured with RCE or αPD-L1 for 24 h at the indicated concentrations. The relative PD-1/PD-L1 blockade activity were measured by PD-1/NFAT luciferase reporter assay. (**F**) Effect of RCE on IL-2 cytokine release in co-cultured cell model. Cell culture media was analyzed to measure IL-2 level by cytokine ELISA. Data are presented as mean ± S.E. of three representative independent experiments. Asterisks indicate significant inhibition of PD-1/PD-L1 binding activity by each test inhibitor as compared with the vehicle-treated group; *** *p* < 0.001, ** *p* < 0.01, * *p* < 0.05, compared with the vehicle group.

To investigate effect of RCE on the interaction between PD-1 and PD-L1, competitive ELISA were performed as previously reported [14]. The results confirmed that RCE dose-dependently interfered the binding of PD-1 and PD-L1 at an indicated concentration in rage from 25 to 800 μg/mL (Figure 1A). The half maximal inhibitory concentration (IC_50_) value of RCE was 83.8 ± 4.7 μg/mL. Antagonist antibody to PD-L1 (αPD-L1) were used as a positive control, with IC_50_ value with 1.69 ± 0.8 μg/mL (Figure 1B).

A luciferase reporter system in co-cultured cell models expressing PD-1 and PD-L1, is one of the well-established bioassay used to determine the PD-1/PD-L1 inhibitors and it has high sensitivity and stability [17]. Prior to the experiment, Cell Counting Kit-8 (CCK) assay was conducted to investigate the non-cytotoxic concentration of RCEs on either cell models (Figure 1C,D). RCEs were cytotoxic at a concentration of 200 and 400 μg/mL for 24 h; therefore, the next experiment was conducted with a non-cytotoxic concentration 100 μg/mL.

As previously reported [14,18], the co-incubation of PD-1/NFAT Jurkat T cells and PD-L1/aAPC CHO-K1 cells resulted in PD-1/PD-L1 interaction, which suppress the T cell receptor (TCR) activation and its downstream signaling, and was determined by the NFAT-luciferase reporter activity. When the PD-1/PD-L1 interaction was disrupted by antibodies or inhibitors, it restores TCR signaling and induces NFAT-mediated luminescence in a co-culture model [18]. Therefore, effect of RCE in PD-1/PD-L1 bioassay was evaluated by the cell-based reporter assay and the results are shown in Figure 1E. Relative luminescence intensities by RCE and αPD-L1 were approximately 2-fold induction, with EC_50_ value of 56.15 ± 14.35 μg/mL (Figure 1E). In order to investigate the ability of RCE to improve T cell activity, the production of IL-2 were analyzed in co-culture systems (Figure 1F). Similar to the result of cell-based PD-1/PD-L1 bioassays, RCE at 100 μg/mL increased the levels of IL-2 up to about 1.8-fold higher than the untreated group. These results suggested that RCE would be a potent agent for PD-1/PD-L1 axis and T cell function.

### 3.2. Anti-Tumor Effect of RCE on Tumor Growth of MC38 Expressing hPD-L1 in Humanized PD-1 Mice

**Figure 2:**
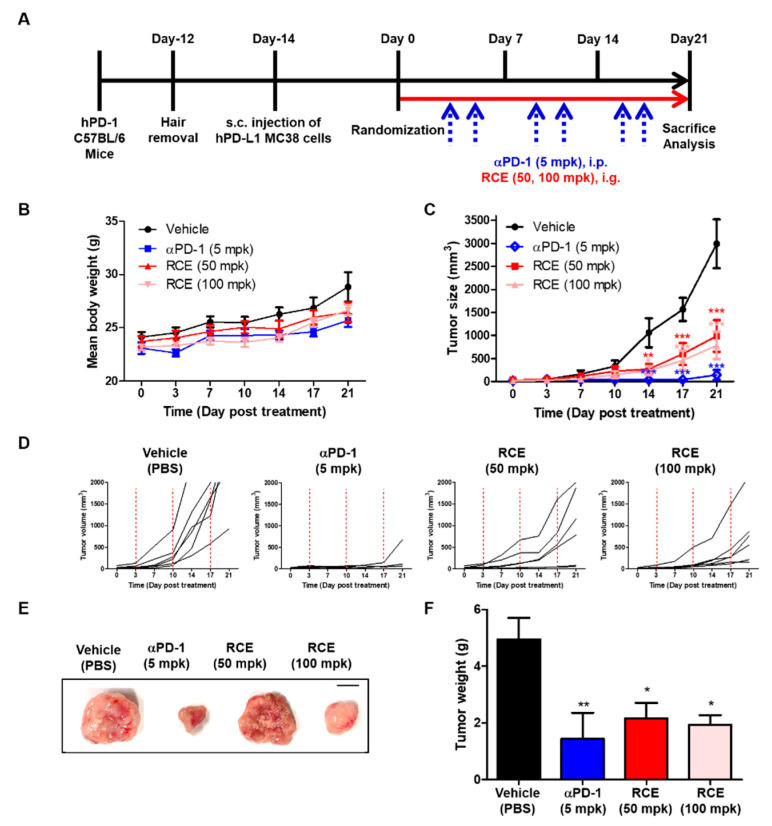
Anti-tumor efficacy of RCE on hPD-L1 MC38 tumor in humanized PD-1 mice. (**A**) Experimental scheme. (**B**) Mean bodyweight. (**C**) Mean tumor growth curves. (**D**) The individual tumor growth over time for plot. MC38 cells expressing hPD-L1 (1.5 × 10^6^ cells/mL) were injected subcutaneously (day-14) into humanized PD-1 mice (3 × 10^5^ cells/200 μL in PBS/mouse). Once tumors became palpable (~100 mm^3^), the tumor-bearing mice were randomly assigned to either a vehicle group or treatment group (*n* = 6). Animals were treated with vehicle (PBS, 10 mL/kg, q.d., i.g.), αPD-1 (5 mpk, b.i.w., i.p.), RCE (50 mpk, q.d., i.g.) and RCE (100 mpk, q.d., i.g.). Tumor mice received an equivalent volume of vehicle (200 μL). On day 21 post administration, all mice were sacrificed for in vivo analysis. (**E**) Representative images of subcutaneous tumor mass on day 21 after treatment (scale bars, 1 cm). (**F**) Mean tumor weight. Statistics were analyzed using two-way ANOVA with Bonferroni post-hoc test; *** *p* < 0.001, ** *p* < 0.01, * *p* < 0.05, compared with the vehicle group.

Considerable attention has been focused on PD-1/PD-L1 therapeutics, and there have been a number of efforts to further develop immuno-oncology (I-O) animal models [19,20]. Recently, humanized immune checkpoint animal models were developed for targeting PD-1/PD-L1 [11] and it was demonstrated that murine PD-L1 deletion and hPD-L1 replacement did not significantly affect tumor cell growth in vitro and in vivo studies [21]. In addition, RCE activities against PD-1/PD-L1 PPI were evaluated based on in vitro assays by utilizing human recombinant proteins and human cell lines, therefore, we selected the hPD-L1 expressing MC38 tumor model for testing the in vivo efficacy of RCE (Figure 2).

To examine the anti-tumor efficacy of RCE, hPD-L1 knock-in MC38 tumor cells were injected subcutaneously (s.c.) into humanized PD-1 mice and RCE and anti-PD-1 were treated orally or intraperitoneally (i.p.) (Figure 2A). Compared with vehicle-treated group (PBS, 10 mL/kg), there were no significant side effects such as severe changes of bodyweight by treatment of RCE and anti-hPD-1 antibody (Figure 2B). The oral administration of RCE (50 and 100 mpk) inhibited the mean tumor volume in a dose-dependent and time-dependent manner (Figure 2C,D). Tumor growth inhibition rates (TGI) of RCE were confirmed as 66.94% and 73.81%, respectively. Moreover, the positive control anti-hPD-1 antibody (5 mpk) exhibited 95.24% TGI on day 21. Consistent results were shown in a representative image of subcutaneous tumor mass (Figure 2E) and the graph of the mean tumor weight (Figure 2F). Collectively, the present results suggest that the oral administration of RCE effectively attenuates tumor growth in humanized PD-1 mice, indicating that RCE could be utilized as a potential candidate agent for cancer therapeutics targeting PD-1 and PD-L1.

### 3.3. Identification of the Main Compound of RCE by HPLC Analysis

**Figure 3:**
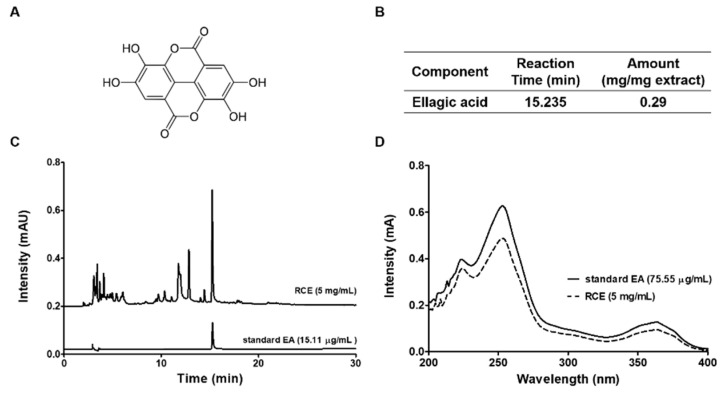
Identification of the main compound of RCE by HPLC analysis. (**A**) Structures of the major compound of RCE. (**B**) The amount of ellagic acid in RCE and retention time. (**C**) High-performance liquid chromatography (HPLC) profiles of ellagic acid from RCE. (**D**) Standard ellagic acid was used for reference compound and monitored at 254 nm.

To examine the content of a main compound of RCE, HPLC analyses were performed with the mobile phase consisting of acetonitrile (ACN) and water, and a C18 column as the stationary phase. According to the Korean Pharmacopoeia, the main component of RCE is ellagic acid (Figure 3A), and of course there are some other phenols and flavonoids, such as cyanidin 3-O-sambubioside, cyanidin 3-O-xylosylrutinoside, cyanidin 3-O-rutinoside, pelargonidin 3-O-rutinoside, delphinidin 3-O-rutinoside, delphinidin 3-O-glucuronide. However, according to previous reports, their content is not high, but very complicated spectra (as shown in Figure 3C). Thus, the most representative component, ellagic acid was chosen as the target of analysis. The calibration curve of the ellagic acid was y = 21323292.21x − 177777 with good linearity (r^2^ = 0.9997) in the tested concentration (1–25 mg/mL). The standards had retention times which included ellagic acid (15.235 min) through the comparison of retention times and UV spectra. The amount of ellagic acid was 0.29 mg/mg of RCE (Figure 3B).

### 3.4. Potent Biological Effects of the Main Compound of RCE

**Figure 4:**
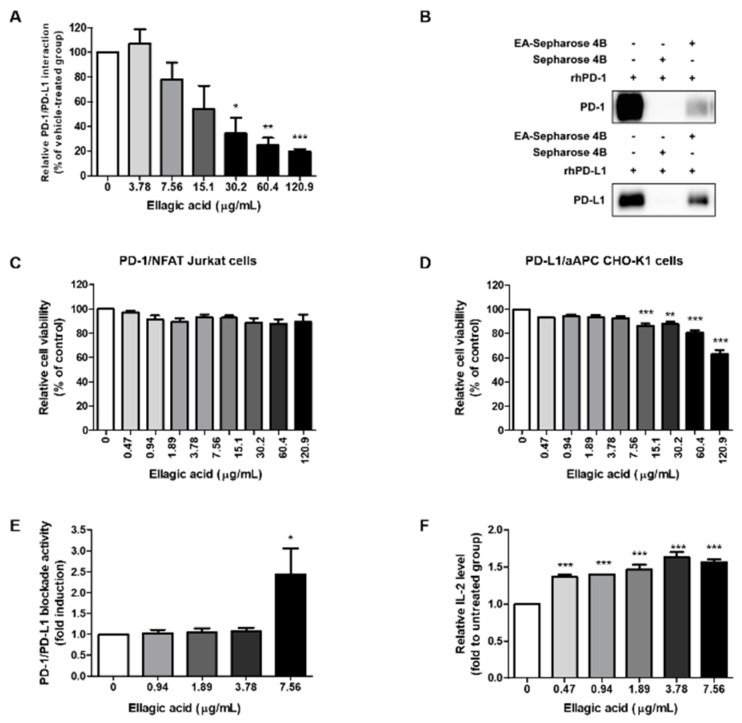
Effect of ellagic acid on PD-1/PD-L1 interaction in vitro. (**A**) Effect of ellagic acid on PD-1/PD-L1 interaction was measured by competitive ELISA. Ellagic acid was treated as indicated concentration (0–120.9 μg/mL). (**B**) Ellagic acid binds to PD-1 and PD-L1. Pull-down assays were performed to detect the binding of ellagic acid with PD-1 or PD-L1. Binding by ellagic acid between human recombinant PD-1 or PD-L1 proteins were confirmed by Western blot using antibodies against PD-1 (upper panel) or PD-L1 (lower panel). Lane 1: Recombinant PD-1 or PD-L1 protein alone; lane 2: each of the proteins were precipitated with Sepharose 4B-alone; lane 3: each of the proteins were precipitated with ellagic acid–Sepharose 4B beads (EA-Sepharose 4B). (**C**,**D**) Effect of ellagic acid on cell viability of (c) PD-1/NFAT Jurkat cells or (**D**) PD-L1/aAPC PD-L1 CHO-K1 cells. The cell viabilities were analyzed using the Cell Counting Kit-8 (CCK) assay. Cells were treated with or ellagic acid as indicated concentrations (0–120.9 μg/mL). (**E**) Effect of ellagic acid on cellular PD-1/PD-L1 blockade activity. PD-1/NFAT Jurkat effector cells and PD-L1/aAPC CHO-K1 target cells were co-cultured with ellagic acid for 48 h at the indicated concentrations (0–7.56 μg/mL). The relative PD-1/PD-L1 blockade activity were measured by PD-1/NFAT luciferase reporter assay. (**F**) Effect of ellagic acid on IL-2 cytokine release in co-cultured cell model. Cell culture media was analyzed to measure IL-2 level by cytokine ELISA. Data are presented as mean ± S.E. of three representative independent experiments. Asterisks indicate significant inhibition of PD-1/PD-L1 binding activity by each test inhibitor as compared with the vehicle-treated group; *** *p* < 0.001, ** *p* < 0.01, * *p* < 0.05, compared with the vehicle group.

As RCE exhibited PD-1 and PD-L1 interaction, we hypothesized that the major compound of RCE, ellagic acid, would affect PD-1 and PD-L1 binding activity. Therefore, competitive ELISA was conducted to investigate the effect of ellagic acid on PD-1/PD-L1 interaction. Results confirmed that ellagic acid blocked PD-1/PD-L1 binding in a dose-dependent manner with IC_50_ value of 22.92 μg/mL (Figure 4A). A pull-down assay using ellagic acid-conjugated sepharose 4B beads showed that ellagic acid directly interacts with recombinant PD-1 (Figure 4B, upper panel). In addition, consistent results were confirmed by ellagic acid–sepharose 4B beads, but not sepharose 4B beads alone, bound to recombinant human PD-L1 (Figure 4B, lower panel). These results indicate that ellagic acid can directly bind to both PD-1 and PD-L1 resulting in interruption of their binding activities. CCK assays showed that ellagic acid did not influence on cell viability up to 7.56 μg/mL in PD-1/NFAT Jurkat cells and PD-L1/aAPC CHO-K1 cells (Figure 4C,D). At non-cytotoxic concentration, ellagic acid blocked PD-1/PD-L1 axis (Figure 4E) and induced IL-2 production in a dose-dependent manner (Figure 4F). These results indicate the possibility that PD-1/PD-L1 blocking effect of RCE were mediated by its main compound, ellagic acid.

## 4. Discussion

Black raspberry is a polyphenol-rich fruit and has been consumed as foods for human health. In addition, unripen fruits of black raspberry (*R. coreanus*) were used as a traditional medicinal plant for kidney disease [22]. Extract of *R. coreanus* (RCE) has reported to possess a variety of biological properties including anti-cancer in a number of in vitro and animal studies [23]. Seeram et al., and Wang et al., reported that RCE reduced the cell proliferation and induced the apoptosis in HT-29, HCT-116, Caco-2, and SW480 colon cancer cell [24,25]. Diets containing black raspberry (2.5, 5, or 10% *w/w*) during 9- or 33-week attenuated azoxymethane (AOM)-induced carcinogenesis by down-regulation of DNA oxidation levels in rats [26]. Another study by Bi X. et al., also showed that diet containing 10% freeze-dried black raspberry for 12 week inhibited tumor incidence and intestinal cell proliferation in Apc1638 +/− and Muc2 −/− mice [27]. In addition, four clinical studies of black raspberry extract have been performed in colorectal cancer (CRC) and Familial Adenomatous Polyposis (FAP) patients up to date [23]. Although these preclinical and clinical studies suggest the possibility of black raspberry as medicinal food products for colorectal cancer, however, the immunomodulatory effect of RCE on T cell activation and antitumor immunity targeting PD-1 and PD-L1 have not been fully understood. As shown in Figure 1, competitive ELISA and PD-1/NFAT reporter assays have confirmed that RCE effectively blocked the interaction between PD-1 and PD-L1. In addition, the in vivo analysis confirmed that RCE attenuates the tumor growth of hPD-L1 knock-in MC38 tumor in humanized PD-1 mice (Figure 2). Therefore, these results suggest that RCE can be a potent immunotherapeutic agent for enhancing T cell activity by inhibiting PD-1/PD-L1 axis.

RCE showed not only antitumor effect via direct antitumor effect by induction of apoptosis and cell cycle arrest in previous study (Figure S1), but also exhibited PD-1/PD-L1 blockade and enhanced T cell function in the current study (Figure 1). These effect may be considered due to the characteristics of natural product that it has composed of multiple compounds with diverse targets and molecular mechanism, resulting in various biological effects [28]. In order to clarify which effect is dominant for overall therapeutic effect, it further needs to clarify the other active components and potential targets of RCE and to predict the potential mechanism of RCE. In addition, new strategies are required by emerging various databases including DrugBank and TCMSP and developing the network pharmacology, as a previous study reported [29].

Black raspberry contains abundant phenolic acids and flavonoids, and the benefit of the black raspberry has been attributed to its various compounds. To date, numerous compounds including 23 phenolic acids have been confirmed from Chinese wild raspberry (*Rubus Chingii Hu*) which is similar species to *R. coreanus* [8]. Among diverse compounds, ellagic acid has been identified as one of the main components of Chinese wild raspberry extract and is associated with various biological activities [30]. Indeed, ellagic acid has reported to have immunoregulatory function and anti-cancer capacity for the prevention of several cancers [31]. Ellagic acid regulates LPS-induced maturation of bone marrow-derived dendritic cells (BMDCs) by the modulation of c-Jun N-terminal kinase (JNK) activity [32]. In addition, ellagic acid was reported to stimulate the apoptosis and cell cycle arrest in HT-29 [33] or HCT-116 [34] human colorectal cancer cells. This research describe that biological activity of black raspberry in treatment of colorectal cancer is closely related to its main compound, ellagic acid. Therefore, HPLC analysis was conducted in order to elucidate whether ellagic acid is a major component of RCE. The chemical analysis of RCE by HPLC shows the existence of ellagic acid at an amount of 0.29 mg/mg of extract (Figure 3). In vitro assay revealed that ellagic acid possesses inhibitory effect on the PD-1/PD-L1 axis blockade and upregulates the release of IL-2 (Figure 4). Therefore, our findings also support that ellagic acid may play an important role in immunomodulatory efficacy of RCE in the present study.

RCE is listed on Korean Food Standards Codex as well as the Korean Pharmacopoeia. This means that RCE could be safe enough for food, on the other hand, it would be effective enough for medicine. So, RCE was registered the ingredient of supplementary food for anti-oxidant and liver function in Korea. Based on our results, RCE can be applied to the diet on cancer patient to enhance anti-cancer immunity via blocking PD-1/PD-L1 interaction.

## 5. Conclusions

The present study describes T cell activation effect of RCE and provide an evidence to discuss the possible antitumor immunity mechanism of RCE by blocking PD-1/PD-L1 axis. In addition, these biological efficacies of RCE seem to be closely involved with the existence of ellagic acid. Based on our results, RCE and its main constitute, ellagic acid, can be utilized as potent immunotherapeutic agents against cancer.

## Supplementary Materials

The following are available online at https://www.mdpi.com/2304-8158/9/11/1590/s1, Figure S1: Effect of RCE or ellagic acid on cell viability of MC38 cells expressing human PD-L1 (hPD-L1 MC38 cells).

## Abbreviations

AOM	Azoxymethane
bw	Body weight
Caco-2	Colon adenocarcinoma isolated from a primary colonic tumor
CRC	Colorectal cancer
EC_50_	A concentration of a drug that gives half-maximal response
PD-1	Programmed cell death protein 1
PD-L1	Programmed death-ligand 1
TCR	T-cell receptor
NFAT	Nuclear factor of activated T cells
mpk	mg/kg
IC_50_	Half maximal inhibitory concentration
IL	Interleukin
i.p.	Intraperitoneal injection
i.g.	Intragastric administration
ICI	Immune checkpoint inhibitor
PPI	Protein-protein interaction
s.c.	Subcutaneously
N/D	Not determined or not reported
wk	Week
