# Supplementary material for: Unripe Black Raspberry (Rubus coreanus Miquel) Extract and Its Constitute, Ellagic Acid Induces T Cell Activation and Antitumor Immunity by Blocking PD-1/PD-L1 Interaction
Source: Foods. 2020 Nov 2;9(11):1590. doi: 10.3390/foods9111590 (PMC7693366; doi:10.3390/foods9111590)
Supplement: Supplementary file 1 [file foods-09-01590-s001.pdf]

S.1. Supplementary data

Supplementary Figure

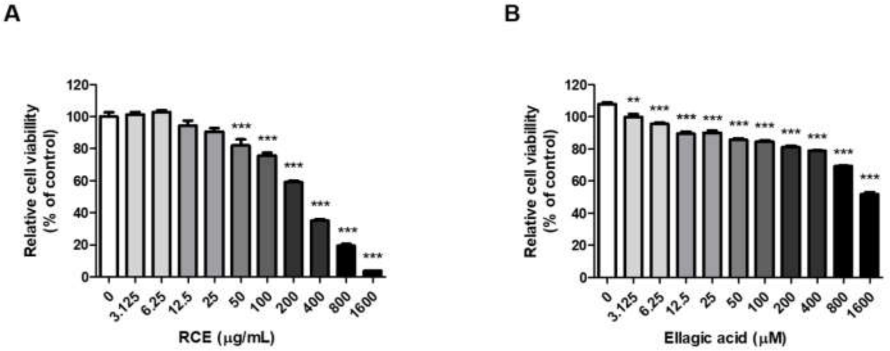

Figure S1. Effect of RCE or ellagic acid on cell viability of MC38 cells expressing human PD-L1 (hPD-L1 MC38 cells). The cell viabilities were analyzed using the Cell Counting Kit-8 (CCK) assay. Cells were treated with RCE or ellagic acid as indicated concentrations for 24 h.

S.2. Supplementary Materials

S.2.1. Cell Culture

Humanized PD-L1 Knock-in MC38 cells (hPD-L1 MC38 cells) derived from C57BL/6 murine colon carcinoma were purchased from Shanghai Model Organisms Center, Inc. (Shanghai, China). The hPD-L1 MC38 cells were maintained in Dulbecco's modified Eagle medium (DMEM) containing 10% and 1% penicillin and streptomycin at 37°C in a 5% CO_2 incubator. For the experiments, Hygromycin B was not included in the medium.

S.2.2. Cell viability assay

Cell viability was measured using Cell Counting Kit-8 (CCK) *assay* according to the distributor's instructions (Dojindo Molecular Technologies, Inc., Rockville, MD, USA). CCK is one of sensitive colorimetric assay for the determination of cell viability. WST-8, highly water-soluble tetrazolium salt, is converted by dehydrogenase activities in cells to a yellow-color formazan dye. Briefly, cells were seeded into 96-well plates at a density of 1×10^4 cells/well and cultured overnight before RCE treatment. Test inhibitors were added to the wells at the indicated concentrations. After incubation for the indicated time, 10 μL of CCK solution was treated for 2 h at 37°C. The amount of the formazan dye, generated by the activities of dehydrogenases in cells, is soluble in the cell culture media and it is directly proportional to the number of living cells. To measure the color density at 450 nm, a microplate reader from Molecular Devices i3 (San Jose, California, USA) was used.

S.2.3. Statistical Analysis

The data were expressed as mean ± the standard error (S.E.) of the mean. Differences in the mean values between the treatment and control groups were analyzed by one-way analysis of variance with Dunnett's post-hoc test for multiple comparisons. GraphPad PRISM software® Version 5.02 (La Jolla, CA, USA) was used for analysis. P-values less than 0.05 were considered significant. Statistical differences are indicated using asterisks.
